# The Divergent Effect of Maternal Protein Restriction during Pregnancy and Postweaning High-Fat Diet Feeding on Blood Pressure and Adiposity in Adult Mouse Offspring

**DOI:** 10.3390/nu10121832

**Published:** 2018-11-28

**Authors:** Dyan Sellayah, Felino R. Cagampang

**Affiliations:** 1 Harborne Building 12A, School of Biological Sciences, University of Reading, Whiteknights, Reading, Berkshire RG6 6AS, UK; 2 Institute of Developmental Sciences, Faculty of Medicine, University of Southampton, Southampton General Hospital (MP887), Tremona Road, Southampton SO16 6YD, UK; f.cagampang@soton.ac.uk

**Keywords:** maternal diet, nutrition, metabolism, hypertension, obesity

## Abstract

Obesity is a growing health crisis of pandemic proportions. Numerous animal and human studies have confirmed that obesity and related metabolic abnormalities, such as insulin resistance and cardiovascular disease, may be programmed during development by adverse maternal nutrition. We previously documented that offspring of female mice who were protein-restricted during pregnancy alone had no alterations to their body weights, but did display a considerable reduction in food intake, a finding which was linked to reduced expression levels of appetite regulatory genes in the hypothalamus. Whether such observations were accompanied by changes in metabolic and phenotypic parameters remained to be determined. Female pregnant MF-1 mice were fed, exclusively during the pregnancy period, a normal protein diet containing 18% casein (C) or an isocaloric protein-restricted diet containing 9% casein (PR). From birth, the lactating dams were fed a normal protein diet. At weaning, offspring were fed either the standard chow which contain 7% kcal fat (C) or high-fat diet (HF, 45% kcal fat). This yielded 4 experimental groups denoted by maternal diet/offspring diet: C/C, C/HF, PR/C, PR/HF. Our results showed that offspring adiposity was significantly increased in HF-fed offspring, and was not affected by the 50% reduction in protein content of the maternal diet fed during pregnancy. Similarly, blood glucose levels were higher in HF-fed offspring, regardless of protein content of the maternal diet. Systolic blood pressure, on the other hand, was significantly increased in both male and female offspring of dams fed the PR diet, and this was exacerbated by a postweaning HF diet. Our results show that maternal protein restriction leads to elevations in systolic blood pressure, which is exacerbated by a postweaning HF-diet. Our present findings suggest that, while changes in offspring adiposity brought about by exposure to maternal protein restriction during pregnancy may be restored by adequate maternal protein content during lactation, the same may not be true for systolic blood pressure, which was similarly impaired, regardless of the timing of maternal low-protein exposure.

## 1. Introduction

Obesity is a risk factor for a plethora of metabolic conditions that greatly affect relative mortality risk, and which collectively impose devastating economic burdens on health systems throughout the world [1]. The now widely accepted Barker hypothesis, which suggests that the *in utero* environment is influential over health in later life, was built around epidemiological observations that birthweight and cardiovascular disease risk in adulthood were highly correlated [2]. Indeed, a multitude of subsequent epidemiological analyses and studies in animal models have confirmed that the *in utero* and early postnatal environments strongly influence adulthood risk of metabolic disease, including cardiovascular disease, fatty liver disease, and insulin resistance [3,4,5]. Both maternal undernutrition and overnutrition have been linked to higher risk of offspring metabolic disease [6]. Various dietary manipulations have been used to model maternal undernutrition during pregnancy, including global caloric restriction, as well as restriction of specific macronutrients [7,8]. Numerous studies using animal models have shown that maternal protein restriction during pregnancy, and/or lactation, program offspring for metabolic dysfunction in later life [9,10,11]. Much of the early work in rodent models of maternal protein restriction was established at the University of Southampton, including our group, and have consistently resulted in hypertension and metabolic dysfunction in the offspring [12,13,14,15,16,17]. Thus, we chose to continue using this established nutritional paradigm of maternal undernutrition. 

Evidence also suggests that, a mismatch, particularly the degree of mismatch, between pre- and postnatal nutritional environments, exacerbates the deleterious effects of adverse nutrition during pregnancy on offspring health. For example, maternal undernutrition, followed by postnatal high-fat diet in rats, is associated with insulin resistance and hyperphagia in offspring [18,19,20]. Gluckman and Hanson have proposed an explanation for such observations, suggesting that the fetus makes adaptive alterations to its biochemical and physiological processes, in order to realign their metabolism to a predicted postnatal environment that is predictive of their *in utero* environment [18]. It is argued that such adaptive responses are critical for promoting survival under periods of perceived nutritional hardships. The majority of animal models of nutritional mismatch between the pre- and postnatal environments have utilised global caloric restriction and subsequent postweaning high-fat diets in rats. We thus sought to assess how maternal protein restriction, exclusively during the pregnancy period, followed by postweaning feeding of a diet high in fat, affected the offspring’s metabolic and cardiac health. 

We have previously reported, in a mouse model, that maternal protein restriction during both pregnancy and lactation was associated with increased body weight and adiposity, which was worsened by a postweaning high-fat diet [17]. This phenotypic pattern was not evident if mice were protein restricted during the pregnancy period alone. We previously documented that female mouse offspring whose mothers were protein-restricted during pregnancy alone had no alterations to their body weights, but did display a considerable reduction in food intake, a finding which was linked to reduced expression levels of appetite regulatory genes in the hypothalamus [21]. Whether such observations were accompanied by changes in metabolic and phenotypic parameters remain to be determined. 

In the present study, we document the effects of maternal protein restriction, exclusively during the pregnancy period, followed by a postweaning high-fat diet on adiposity, glucose homeostasis, and systolic blood pressure. 

## 2. Materials and Methods

### 2.1. Animals and Dietary Challenges

All animal procedures were carried out at the University of Southampton, in accordance with the regulations of the United Kingdom Animals (Scientific Procedures) Act, 1986, and were conducted under Home Office Project Licence number 70/6457. The study received institutional approval from the University of Southampton Biomedical Research Facility Research Ethics Committee. Female MF-1 mice were individually housed under a 12 h light–dark cycle, and given free access to drinking water for the duration of the study. At 8–10 weeks of age, females were time-mated, and upon confirmation of pregnancy (presence of vaginal plug), were randomly assigned to one of two dietary regimen groups. One group (*n* = 10) was fed a normal protein chow (C) diet consisting of 18% casein, and the other group (*n* = 10) fed an isocaloric protein-restricted diet consisting of 9% casein (PR). These diets were specially formulated to be isocaloric. Pregnant dams were fed the PR or C diets throughout pregnancy. At birth, litter size was standardised to 8 pups per litter. From birth until the end of lactation (3 weeks), at which point offspring were weaned, all lactating dams were given the standard chow (C) diet. From weaning to the end of the study, when the offspring were 16 weeks of age, they were either fed the standard chow (C) or a high-fat (HF) diet. The HF diet contained 45% kcal fat, whilst the C diet had 7% kcal fat, with casein content of 26% and 15%, respectively. We have previously used this HF diet to bring about an obese phenotype in mice [5]. This experimental paradigm yielded 4 offspring groups based on maternal/offspring diet: C/C, PR/C, C/HF, PR/HF. The nutritional composition of the diets is given in Table 1. 

### 2.2. Adiposity

Total body fat was calculated by determining the cumulative weight of the major individual fat depots (gonadal, inguinal, interscapular, retroperitoneal, and perirenal). The individual and total fat depot weights were expressed as a percentage of total body fat. 

### 2.3. Plasma Leptin Levels

A mouse leptin radioimmunoassay kit was used to determine plasma leptin levels in offspring. Calibrators (0.5, 1, 2, 5, 10, 20, and 50 μg/L) or specimens were pipetted in duplicate into tubes at 100 μL each, and combined with an antibody against leptin (100 μL). After incubation for at room temperature for 18–24 h (disequilibrium assay format), 100 μL of ^125^I-tracer was added to each tube and incubated for a further 18–24 h. Cold precipitating antibody (1.0 mL; anti-guinea pig IgG, raised in goats) was added to all tubes, which were subsequently incubated for 20 min at 4 °C, in order to precipitate the antibody/leptin complex. Following centrifugation for 20 min at 2500× *g* at 4 °C, visible pellets were obtained. Radioactivity of the pellets was counted. Log values of calibrators were plotted vs. the calibrator-bound counts/zero calibrator-bound counts (*B*/*B*o), to generate a curve for calculation of unknowns. 

### 2.4. Systolic Blood Pressure and Fasting Blood Glucose

Systolic blood pressure was determined by tail-cuff plethysmography, as previously described [22]. Measurements were conducted in a heated room, in order to obtain optimal blood flow, and were conducted at the same time each day the measurements were performed. All animals were allowed to acclimatise to the procedure prior to each recorded session.

At termination of the study at 16 weeks of age, animals were fasted overnight, and sacrificed the following day by cervical dislocation. Blood samples were collected via cardiac puncture and blood glucose was measured using a blood glucose meter (Accu-Chek Aviva, Roche, Penzberg, Germany). 

### 2.5. Statistical Analysis

All values are presented as mean ± SEM. All data were analysed statistically using one-way analysis of variance (ANOVA) followed by the Tukey–Kramer test for comparisons, where appropriate. Statistical significance was assumed if *p* < 0.05. All analysis was performed using Prism (GraphPad Software Inc., San Diego, CA, USA) statistical programs.

## 3. Results

### 3.1. Adiposity and Plasma Leptin Levels

**Figure 1:**
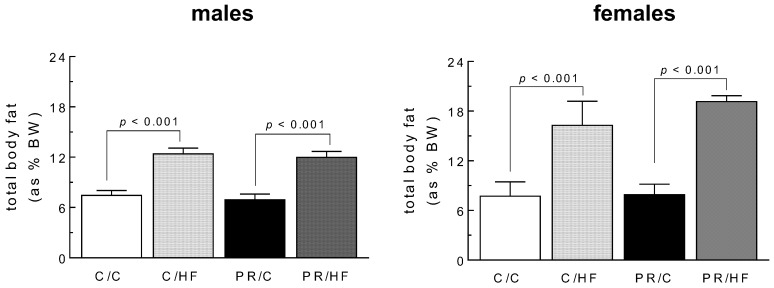
Total body fat (expressed as % of body weight) in male and female offspring from dams on standard on standard chow (C) or protein restricted (PR) diet during pregnancy. Weaned offspring were then fed either a high fat (HF) or C diet to adulthood, thus generating the dam/offspring dietary group: C/C, C/HF, PR/C and PR/HF. All values are reported as means ± SME (*n* = 7–11 per group) and analyzed using a one-way ANOVA with Turkey-Kramer *post hoc* test.

**Figure 2:**
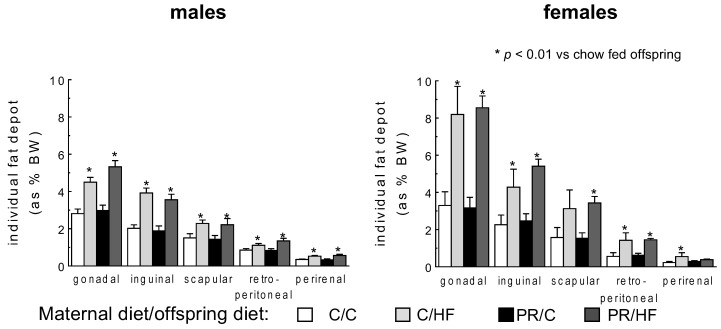
Weight of individual fat depots (expressed as % of total body weight) in male and female offspring from dams on standard on standard chow (C) or protein restricted (PR) diet during pregnancy. Weaned offspring were then fed either a high fat (HF) or C diet to adulthood, thus generating the dam/offspring dietary group: C/C, C/HF, PR/C and PR/HF. All values are reported as means ± SME (*n* = 7–11 per group) and analyzed using a one-way ANOVA with Turkey-Kramer *post hoc* test.

Total body fat was elevated by 50% in HF-fed male and female offspring, compared to their counterparts fed the standard chow diet (Figure 1). On the other hand, mothers’ dietary protein content had no effect on offspring adiposity (*p* < 0.001). A similar pattern was observed in individual fat depots, wherein postweaning HF-diet in offspring had a profound effect on adiposity in both males and females, increasing fat depot weights by between 30% to 120%, compared to chow-fed offspring (Figure 2). There were no statistically significant differences between offspring of protein-restricted mothers and those from mothers fed a normal protein diet. Thus, maternal protein restriction was not associated with changes in adiposity in offspring.

### 3.2. Plasma Leptin Levels and Fasting Blood Glucose

**Figure 3:**
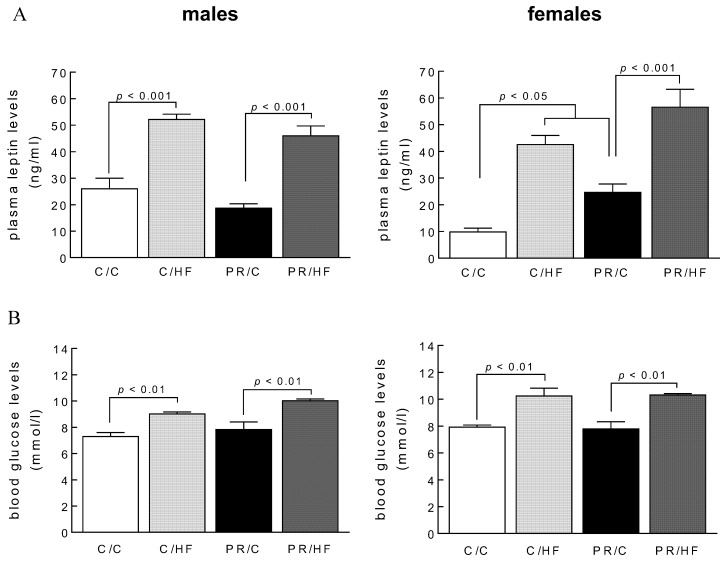
(**A**) Plasma leptin levels and (**B**) Blood glucose levels in male in male and female offspring from dams on standard on standard chow (C) or protein restricted (PR) diet during pregnancy. Weaned offspring were then fed either a high fat (HF) or C diet to adulthood, thus generating the dam/offspring dietary group: C/C, C/HF, PR/C and PR/HF. All values are reported as means ± SME (*n* = 7–11 per group) and analyzed using a one-way ANOVA with Turkey-Kramer *post hoc* test.

Plasma leptin levels were elevated by two-fold in male and female HF-fed offspring, compared to their chow-fed counterparts (*p* < 0.001) (Figure 3A). We observed that chow-fed female offspring of protein-restricted mothers had higher leptin levels compared to chow-fed female offspring of mothers fed a normal protein diet during pregnancy (PR/C vs. C/C *p* < 0.05). Fasting blood glucose levels were elevated by 15% in both male and female HF-fed offspring, compared to offspring fed a chow diet postweaning (*p* < 0.01). On the other hand, offspring blood glucose levels were unaffected by maternal protein content during pregnancy (Figure 3B). 

### 3.3. Systolic Blood Pressure and Heart Weights

**Figure 4:**
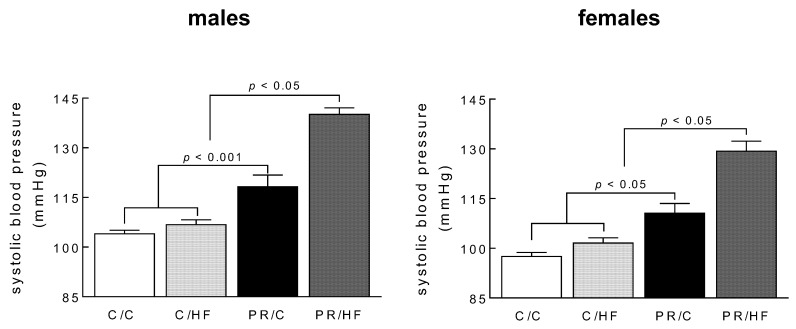
Systolic blood pressure in male and female offspring from dams on standard chow (C) or protein restricted (PR) diet during pregnancy. Weaned offspring were then fed either a high fat (HF) or C diet to adulthood, thus generating the dam/offspring dietary group: C/C, C/HF, PR/C and PR/HF. All values are reported as means ± SME (*n* = 7–11 per group) and analyzed using a one-way ANOVA with Turkey-Kramer *post hoc* test.

Systolic blood pressure was higher in both male (*p* < 0.001) and female (*p* < 0.05) chow-fed offspring of protein-restricted mothers, compared to the chow-fed counterparts of mothers fed normal protein diet during pregnancy (Figure 4). Systolic blood pressure was further elevated in HF-fed male and female offspring of protein-restricted mothers (*p* < 0.05), compared with the PR/C offspring, respectively. These data suggest that, unlike in adiposity, there is an interaction between maternal and postweaning nutrition to influence offspring systolic blood pressure. 

## 4. Discussion

Our results demonstrate that maternal protein restriction during pregnancy alone has no effect on offspring adiposity, irrespective of whether the offspring were fed a high-fat diet or chow diet. This is surprisingly different from our previous findings, wherein maternal protein restriction was extended to include the lactation period [17]. In this previous study, maternal protein restriction, during pregnancy and lactation, led to increased adiposity in male offspring, an observation which was exacerbated by a postweaning HF diet. Thus, it appears that the window of exposure to maternal protein restriction is influential over adiposity of offspring. Studies suggest that adequate nutrition during lactation may reverse the adverse effects of maternal undernutrition during pregnancy, on offspring adiposity and metabolic function, although further studies are warranted to examine this in more detail [23]. 

Our previous studies have shown that maternal protein restriction during pregnancy, followed by postweaning HF diet, does not lead to any statistically significant differences in body weight trajectory, however, it does lead to a 20% reduction in food intake, compared to HF-fed offspring of dams fed normal protein levels during pregnancy [17,21]. The present study, however, reveals that maternal protein restriction, confined to only the pregnancy period, had no effect on offspring adiposity. This suggests that PR/HF male mouse offspring have increased energy efficiency and reduced appetite, which may be adaptive mechanisms to defend against weight gain. We have previously reported in HF-fed offspring of dams fed a low-protein diet during pregnancy and lactation, that energy expenditure and the expression of brown adipose tissue genes are significantly reduced, compared with their HF-fed counterparts from chow-fed control dams [17]. Whether or not a similar mechanism is involved in increased energy efficiency in HF-fed offspring of dams who were protein restricted exclusively during the pregnancy period, requires further investigation. Studies have shown that adipose tissue dysfunction, caused by adverse maternal nutrition during pregnancy, can be reversed by restoration of adequate nutrition during the lactation period [24]. Moreover, adverse maternal nutrition during the lactation period alone has been shown to alter offspring brown adipose function [25]. Thus, taken together, these studies suggest that adverse nutrition in the lactation period is critical for the manifestation of alterations in adipose tissue amount and function. 

Despite finding no differences in adiposity in offspring of the current study, we did observe increased plasma leptin levels in chow-fed female offspring of protein-restricted dams. This observation was absent in males. Elevated leptin has been shown to reflect leptin resistance, in which obese rodents have higher levels of circulating leptin proportional to increased fat mass (leptin is produced and secreted by adipocytes) [26]. However, we did not observe any increases in adiposity of female PR/C offspring. The fact this observation was sex-specific may point to the influence of sex hormones, which have been shown to mediate sex-specific alterations in feeding behaviours, potentially through modulation of the central leptin signalling [27,28]. It is noteworthy, however, that our previous study has documented that there are no significant alterations to total energy intake in female PR/C offspring, compared with control animals. 

While we observed no differences in adiposity in either chow or HF-fed offspring of protein-restricted dams, we did observe that maternal protein restriction was associated with increased systolic blood pressure, which was further exacerbated by a postweaning HF diet. This observation was seen in both male and female offspring, and reflects the observations made in our previous study, documenting the effects of maternal protein restriction during both the pregnancy and lactation period. Thus, contrary to observations in adiposity, changes in systolic blood pressure in the offspring appear not to be influenced by window of exposure/timing of the maternal low-protein insult, such that maternal protein restriction during the pregnancy period alone, and during both the pregnancy and lactation period, produce similar effects on systolic blood pressure. Our results suggest that maternal protein restriction, in utero, programs hypertension in the offspring, and that this response may not be reversed, even with adequate maternal nutrition during the lactation period. These data are in agreement with previous findings in rats, where both in utero low-protein exposure, as well as global caloric restriction, led to elevations in systolic blood pressure [29,30]. While it is well established that obesity and weight gain can promote hypertension, it is clear that adiposity is not a causal factor in the elevated systolic blood pressure in offspring of protein-restricted mothers. More direct effects of adverse maternal nutrition on offspring cardiovascular function are likely to explain the offspring phenotype in the present study. To this end, hypertension has been documented as early as 4 weeks of age, in rats, before the onset of obesity or elevated adiposity, and appears to be maintained throughout adult life, regardless of postnatal nutrition [31]. The mechanistic basis for programmed hypertension in response to maternal undernutrition is still controversial, and may reflect alterations to multiple physiological systems that are interconnected, including an altered renin–angiotensin system [14], glucocorticoid regulation [32], and vascular endothelial dysfunction [33]. 

The results of the present study, and that of our previously published work, show that while elevations in offspring systolic blood pressure induced by maternal protein restriction are unaffected by the timing of when maternal protein restriction was imposed, namely, during pregnancy alone or during both pregnancy and lactation, offspring adiposity is influenced by timing of the exposure to maternal protein restriction. Our results suggest that the window of exposure to maternal protein restriction is important for adiposity and adipose tissue function, but not for cardiovascular function, at least in terms of programmed hypertension. This may reflect the fact that, while the major organs such as heart, kidney, and liver are fully formed and functional by birth, the adipose depots, particularly white adipose depots, are not fully developed until the end of lactation, and may thus be sensitive to dietary alterations only during the lactation period [34,35]. Similar observations have been made in studies on muscle tissue, in which adverse maternal nutrition during pregnancy has been shown to permanently and irreversibly alter muscle function in offspring, leading to altered glucose homeostasis [36,37]. 

In conclusion, we have demonstrated that maternal protein restriction during pregnancy alone, followed by postweaning HF diet, has no effect on offspring adiposity, in spite of a reduced energy intake in male offspring, as reported in our previous study. This lack of effect of maternal protein restriction on adiposity may be related to the window of exposure to maternal protein restriction, as protein restriction during pregnancy and lactation, combined, is associated with significant changes in offspring adiposity. Our results also reveal that hypertension is programmed by maternal protein restriction during pregnancy, and this effect is unrelated to changes in adiposity. These findings mirror that of our previous results, when maternal protein restriction was imposed during both the pregnancy and lactation period, and suggest that the window of exposure may be less important for the programming of cardiovascular dysfunction than adiposity, given that restoring protein content in the maternal diet during lactation rescues the latter, but not the former. 

## Figures and Tables

**Table 1:** Nutritional composition of diets used.

Diet	Sugars (% w/w)	Fat (% w/w)	Fat (% kcal)	Protein (% w/w)	Energy (MJ/kg)
**Maternal Diets**					
Normal Protein Chow (C)	21	10	21	18	18.39
Protein-Restricted (PR)	24	10	21	9	18.27
**Offspring Diets**					
Standard Chow (C)	7	3	7	15	14.74
High-Fat (HF)	10	22	45	26	18.97
